# BMP2 signalling activation enhances bone metastases of non‐small cell lung cancer

**DOI:** 10.1111/jcmm.15702

**Published:** 2020-08-04

**Authors:** Fei Huang, Yaqiang Cao, Gui Wu, Junying Chen, CaihongWang, Wanzun Lin, Ruilong Lan, Bing Wu, Xianhe Xie, Jinsheng Hong, Lengxi Fu

**Affiliations:** ^1^ Central Laboratory First Affiliated Hospital Fujian Medical University Fuzhou China; ^2^ Fujian Platform for Medical Research at First Affiliated Hospital of Fujian Medical University Fuzhou China; ^3^ Fujian Key Laboratory of Individualized Active Immunotherapy Fuzhou China; ^4^ Key Laboratory of Radiation Biology of Fujian Province Universities Fuzhou China; ^5^ CAS Key Laboratory of Computational Biology CAS‐MPG Partner Institute for Computational Biology Shanghai Institute of Nutrition and Health Shanghai Institutes for Biological Sciences University of Chinese Academy of Sciences Chinese Academy of Sciences Shanghai China; ^6^ Department of Orthopedics First Affiliated hospital Fujian Medical University Fuzhou China; ^7^ Department of Radiation Oncology First Affiliated hospital Fujian Medical University Fuzhou China; ^8^ Department of Chemotherapy First Affiliated hospital Fujian Medical University Fuzhou China

**Keywords:** BMP2 signalling, bone metastases, NSCLC, osteoblastic mechanism, osteolytic mechanism

## Abstract

Distant metastases occur when non‐small cell lung cancer (NSCLC) is at late stages. Bone metastasis is one of the most frequent metastases of NSCLC and leads to poor prognosis. It has been reported that high expression of BMP2 in NSCLC correlates with poor survival, but whether BMP2 contributes to NSCLC bone metastasis remains largely unknown. The activation of BMP signalling is found in metastatic bone tumours of mice Lewis lung carcinoma and predicts poor survival in human NSCLC. BMP2 signalling activation can enhance bone metastasis of Lewis lung carcinoma. Moreover, BMP2 secreted by stroma fibroblasts can promote the migration and invasion of NSCLC cells. Besides, in combination with pre‐osteoblast and LLCs, BMP2 could enhance the differentiation of macrophages into osteoclasts to play roles in the osteolytic mechanism of NSCLC bone metastasis. Interestingly, NSCLC cells can also enrich BMP2 to pre‐osteoblasts to function in the osteoblastic mechanism. Our results firstly demonstrate the detailed mechanisms about what roles BMP2 signalling play in enhancing NSCLC bone metastases. These findings provide a new potential therapy choice for preventing bone metastases of NSCLC via the inhibition of BMP2 signalling.

**Highlights**

- BMP signalling is activated in mice NSCLC bone metastases,
- BMP2 signalling correlates with poor survival in human NSCLC.
- BMP2, majorly derived from stroma fibroblast cells, enhances migration and invasion of NSCLC cells.
- BMP2 signalling is associated both osteolytic and osteoblastic mechanisms of NSCLC bone metastases.

## 1 INTRODUCTION

Non‐small cell lung cancer (NSCLC) is the leading cause of cancer death worldwide.^1^, ^2^, ^3^ Distant metastasis frequently occurs when NSCLC is at late stages, which may result in poor prognosis. It has been reported that the median survival time of patients with NSCLC metastases is between 14 and 17 months.^4^ Following breast cancer and prostate cancer, NSCLC is the third most common cancer type that can lead to bone metastasis. About 30%‐40% NSCLC patients are diagnosed with bone metastases at late stages.^5^

The high expression of bone morphogenic protein 2 (BMP2) has been reported in NSCLC.^6^, ^7^, ^8^, ^9^, ^10^ The activation of BMP2 signalling is also found to enhance cell proliferation, migration, invasion and lung metastases of lung adenocarcinoma.^9^, ^11^, ^12^ In addition, the high expression of BMP2 in the stroma indicates poor prognosis in NSCLC.^13^ However, the roles that BMP2 plays in NSCLC bone metastases still remain largely unknown and the detailed mechanisms are unclear.

Usually, according to the effect of cancers on normal bone remodelling, bone metastases can be further classified into osteolytic, osteoblastic or mixed subtypes.^14^, ^15^ Osteolytic, which is characterized by bone resorption induced by activated osteoclasts, has been reported to play roles in NSCLC bone metastases.^16^, ^17^ Parathyroid hormone‐related peptide (PTHrP) and receptor activator of nuclear factor‐kappa‐B ligand (RANKL), which play significant roles in osteoclasts activation and bone metastases,^17^, ^18^ are found to be associated with NSCLC bone metastases.^19^, ^20^ Moreover, miRNA‐33a, targeting PTHrP, is confirmed to reduce bone resorption in NSCLC.^20^ In addition, matrix metalloproteinase (MMPs) and their upstream signalling pathways, like transforming growth factor (TGF‐β), Wnt, chemokine (C‐X‐C Motif) receptor 4 (CXCR4) and nuclear factor kappa‐B (NFκB), may also contribute to NSCLC bone metastases via MMPs.^5^, ^17^, ^21^, ^22^, ^23^, ^24^

The osteoblastic mechanism is usually found in prostate cancer.^25^ Osteogenesis‐associated factors, such as TGF‐β, BMPs and endothelin‐1, play important roles in immature bones formation within tumours.^18^, ^26^, ^27^ Although the preliminary osteolytic mechanism of NSCLC bone metastases has been revealed, it is still unclear about whether the osteoblastic mechanism also occurs in NSCLC bone metastases.

Here, we investigate how BMP2 signalling activation functions in bone metastases of NSCLC. In this study, BMP signalling is found to be activated in bone metastasis of mice Lewis lung carcinoma and correlates with poor survival in human NSCLC. BMP2, derived from the stroma fibroblasts, promotes the migration and invasion of NSCLC cells. Besides, BMP2 plays role in both osteolytic mechanism and osteoblastic mechanism of Lewis lung carcinoma bone metastasis. Taken together, inhibition of BMP2 signalling may be a new therapy choice for the NSCLC bone metastasis patients.

## 2 MATERIALS AND METHODS

### 2.1 Antibodies and reagents

Antibodies were used in this study: monoclonal anti‐Smad1/5 (Cell Signaling Technology, 6944); anti‐pSmad1/5 (Cell Signaling Technology, 9516); anti‐Smad1 (Abcam, ab63356); anti‐Keratin 18 (CK18) (MutiSciences, 70‐ab36769‐050); anti‐p‐Akt (Cell Signaling Technology, 4060); anti‐Akt (Cell Signaling Technology, 2920); anti‐p‐Erk (Cell Signaling Technology, 4370); anti‐Erk (Cell Signaling Technology, 4695); anti‐Osteocalcin (OCN) (Biorbyt, orb259644); anti‐Ki67‐FITC (fluorescein isothiocyanate) (Biolegend, 652 409); anti‐BMP2 (Servicebio, GB11252); TRITC (Tetramethylrhodamine)‐conjugated anti‐Rabbit antibody (abclonal, AS040); FITC‐conjugated anti‐Mouse antibody (abclonal, AS001) and anti‐β‐Actin (Sigma, A1978). Reagents: BMP2 (R&D, 355‐BM‐100); 4',6‐diamidino‐2‐phenylindole (DAPI) (Solarbio, C0060) and carboxyfluorescein succinimidyl amino ester (CFSE) (eBioscience, 65‐0850‐84). Tris‐HCl, NaCl and other chemicals were from Sigma.

### 2.2 Cells

NSCLC cell lines: Lewis lung cells, NCIH‐1373, A549; the macrophage cell line: Raw 264.7, and the pre‐osteoblast cell line: MC3T3‐E1 cells were from ATCC. The ATCC number of Lewis lung cells (LLCs) was CRL‐1642. The ATCC number of NCI‐H1373 cells was CRL‐5866. The ATCC number of A549 cells was CCL‐185. The ATCC number of Raw264.7 cells was TIB‐71. The ATCC number of MC3T3‐E1 cells was CRL‐2594. Lewis lung cells and NCIH‐1373 cells were cultured in RMPI1640 (Invitrogen, Carlsbad, CA, USA) with 10% foetal bovine serum (FBS) (Hyclone, Utah, USA); A549 and Raw 264.7 cells in DMEM (Invitrogen) with 10% FBS (Hyclone) and MC3T3‐E1 cells in α‐MEM (Invitrogen) with 10% FBS (Hyclone). Mouse embryonic fibroblast (MEF) cells were extracted from 13.5 days embryos of C57BL/6 mice and cultured in DMEM (Invitrogen) with 10% FBS (Hyclone). The MEF cells used were less than five passages.

### 2.3 Mice

C57BL /6 mice, female, 6‐8 weeks, used in this study were bred and maintained in a specific pathogen‐free animal facility at Fujian Medical University. Mice were killed with carbon dioxide asphyxiation. All animal experiments were approved by the Animal Ethical Committee of Fujian Medical University (2018‐039).

### 2.4 Lewis lung carcinoma metastasis

1 × 10^6^ LLCs were injected into the tail veins of C57/BL6 mice to make the model of metastasis. LLCs usually tend to metastasize to the lungs and bones. 35 days after injection, mice were killed with carbon dioxide asphyxiation to get the metastatic tissues.

1 × 10^6^ the vehicle or 20 ng/mL BMP2 pre‐treated LLCs were injected into the left lung lobes of C57BL/6 mice to establish the orthotopic model. LLCs usually tend to colonize in lungs and bones. 35 days after injection, mice were killed with carbon dioxide asphyxiation and tissues were harvested for the following analyses.

For the invasive model, 1 × 10^5^ the vehicle or 20 ng/mL BMP2 pre‐treated LLCs were injected into the left hind legs of C57BL/6 mice subcutaneously. To retain the BMP2 signalling activation in the hind leg muscles, 3 μg/kg vehicle or BMP2 was further injected into the left hind leg subcutaneously per week. 35 days after injection, mice were killed with carbon dioxide asphyxiation and tissues were harvested for the following analyses.

### 2.5 Haematoxylin and eosin stain (HE)

The tissues were embedded in paraffin to be cut into 2.5μm tissue sections. Tissue sections were dewaxed with xylene. Then, sections were rehydrated with 100% −95% −75% alcohol gradients. Haematoxylin was stained for 20 minutes, and then, sections were differentiated with 1% hydrochloric acid for 30s. Then, after 15 minutes of PBS blue staining, eosin was stained for 3 minutes. After rinsing, sections were dehydrated with a gradient of 95% to 100% alcohol, and cleared the sections with xylene for two times. Then, sections were mounted with a neutral resin. Photographs were taken by Olympus microscope BX53.

### 2.6 Immunohistochemistry

The tissues were embedded in paraffin to be cut into 2.5 μm tissue sections. Tissue sections were dewaxed with xylene. Then, sections were rehydrated with 100% −95% −75% alcohol gradients. After antigens were retrieved, the sections were incubated with the primary antibodies overnight at 4°C. Then, the sections were incubated with secondary antibodies after briefly washed with phosphate buffer saline (PBS) at room temperature for 2 hours. Finally, the sections were incubated with HRP (horseradish peroxidase)‐conjugated streptavidin for 10 minutes before diamino benzidine (DAB)/H_2_O_2_ was used as the substrate for detection reaction. Photographs were taken by Olympus microscope BX53.

### 2.7 Immunofluorescence

For tissue sections, the protocol was the same with immunohistochemistry before the primary antibodies incubated. For cells, cells were fixed with 4% paraformaldehyde (Sigma) for 20 minutes. Then, cells were permeated with 0.5% Triton X‐100 for five minutes at room temperature. 10% bovine serum albumin (BSA) was used to block the cells for 20 minutes at room temperature. After that, cells were incubated with primary antibodies overnight at 4°C. At the second day, cells and sections were incubated with TRITC‐ or FITC‐conjugated secondary antibody for one hour at room temperature. DAPI (Solarbio, C0060) was used to stain the nucleus. Images were taken by Zessis LSM 800 Laser scanning confocal microscope.

### 2.8 Immunoblotting

Cells and tissues were lysed with TNE buffer (10 mM Tris‐HCl, 150 mM NaCl, 1 mM ethylene diamine tetraacetic acid (EDTA), 0.5% nonidet P 40 (NP40) and pH = 7.5). For immunoblotting assay, cell lysates were mixed with 4 × loading buffer (40 mM Tris‐HCl, 200 mM dithiothreitol (DTT), 4% sodium dodecyl sulphate (SDS), 40% glycerol, 0.032% bromophenol blue, pH = 8.0). The samples were run with 4% stacking gel and 10% separating gels. Then, proteins on the gels were transferred to nitrocellulose filter membranes for antibodies incubated. The membranes’ exposure was done with Thermo Pierce ECL and FluorChem E (Protein Simple).

### 2.9 CFSE‐labelled LLC cells

LLCs were resuspended in 1 mL PBS with 2.5 μM CFSE. LLCs dyed in CFSE solution were vortex in 37℃ water bath for 10 min. Cells were washed with complete RPMI‐1640 culture medium for twice before cultured.

### 2.10 Cell migration assays

1 × 10^4^ cells in culture media without FBS were placed on the upper layer of Corning cell culture insert with polycarbonate membrane (Transwell^@^, 8.0 μm pore size, Corning). The complete culture media with or without 20 ng/mL BMP2 were placed below the cell permeable membrane. Following an incubation period (24 hours) in 37 ℃, 5% CO_2_, the cells that had migrated through the membrane were stained with 0.1% crystal violet and counted.

### 2.11 Cell invasion assays

Corning cell culture insert with polycarbonate membrane (Transwell^@^, 8.0 μm pore size, Corning) were pre‐treated with 10:1 DMEM and matrigels (BD BioSciences). 1 × 10^5^ cells in culture media without FBS were placed on the upper layer of the diluted matrigels. The complete culture media with or without 20 ng/mL BMP2 were placed below the cell permeable membrane. Following an incubation period (48 hours) in 37℃, 5% CO_2_, the cells that had migrated through the membrane were stained with 0.1% crystal violet and counted.

### 2.12 Cell proliferation and apoptosis assays

LLCs or MC3T3‐E1 cells were placed on the upper layer of Corning cell culture insert with polycarbonate membrane (Transwell^@^, 3.0μm pore size, Corning). MC3T3‐E1 cells were cultured in media with or without 200 ng/mL BMP2 for 24 hours. MC3T3‐E1 cells were harvested and stained with the Ki67‐FITC antibody (eBioscience, 11‐5698‐80) or the Allophycocyanin (APC) Annexin V Apoptosis Detection Kit with 7‐AAD (Biolegend, 640 930). Flow cytometric analysis was performed with BD FACS C6 Flow Cytometer. The results were analysed by the software FlowJo 7.6.1.

### 2.13 Alkaline phosphatase (ALP) staining

LLCs or MC3T3‐E1 cells were placed on the upper layer of Corning cell culture insert with polycarbonate membrane (Transwell^@^, 3.0 μm pore size, Corning). MC3T3‐E1 cells below were cultured in media with or without 200 ng/mL BMP2 to be differentiated for seven days. ALP staining was conducted with BCIP/NBT Alkaline Phosphatase Color Development Kit (Beyotime).

### 2.14 Alizarin red staining

LLCs or MC3T3‐E1 cells were placed on the upper layer of Corning cell culture insert with polycarbonate membrane (Transwell^@^, 3.0μm pore size, Corning). MC3T3‐E1 cells below were cultured in media with or without 200 ng/mL BMP2 to be differentiated for 14‐21 days. After cells were fixed with 95% Ethanol for five minutes, 1% Alizarin red (pH = 4.2) was stained for 15 minutes.

### 2.15 Tartrate‐resistant acid phosphatase (TRAP) staining

Murine pre‐osteoclast RAW 264.7 cells (3 × 10^4^ cells/well) were seeded directly into the wells of the 6‐well co‐culture plates, and murine pre‐osteoblast MC3T3‐E1 cells (3 × 10^4^ cells/well) were seeded into the Corning cell culture inserts with polycarbonate membrane (Transwell^@^, 0.4μm pore size) of the co‐culture 6‐well plates. After MC3T3‐E1 cells were attached to the membrane of the inserts, lung cancer cells (3 × 10^4^ cells/well) were added on top of MC3T3‐E1 cell layer in triplicate and treated with 20 ng/mL BMP2 or vehicle. The co‐culture assays were performed in DMEM medium supplemented with 10% FBS and changed every two days. TRAP staining was performed on day 6 using a leucocyte acid phosphatase kit (Sigma, 387A). TRAP^+^‐multinucleated cells were scored as mature osteoclasts and quantified.

### 2.16 ELISA assay

Tumour tissues were homogenized in PBS to be samples. All samples originated from tumours were quantified to the concentration 10 mg/mL by BCA assay. The BCA kit was from Byeotime (P0009). Samples from tumours or supernatant from cell cultures were subjected to BMP2 ELISA assay (R&D, DBP200).

### 2.17 RNA‐SEQ and bioinformatics analysis

**Figure 1:**
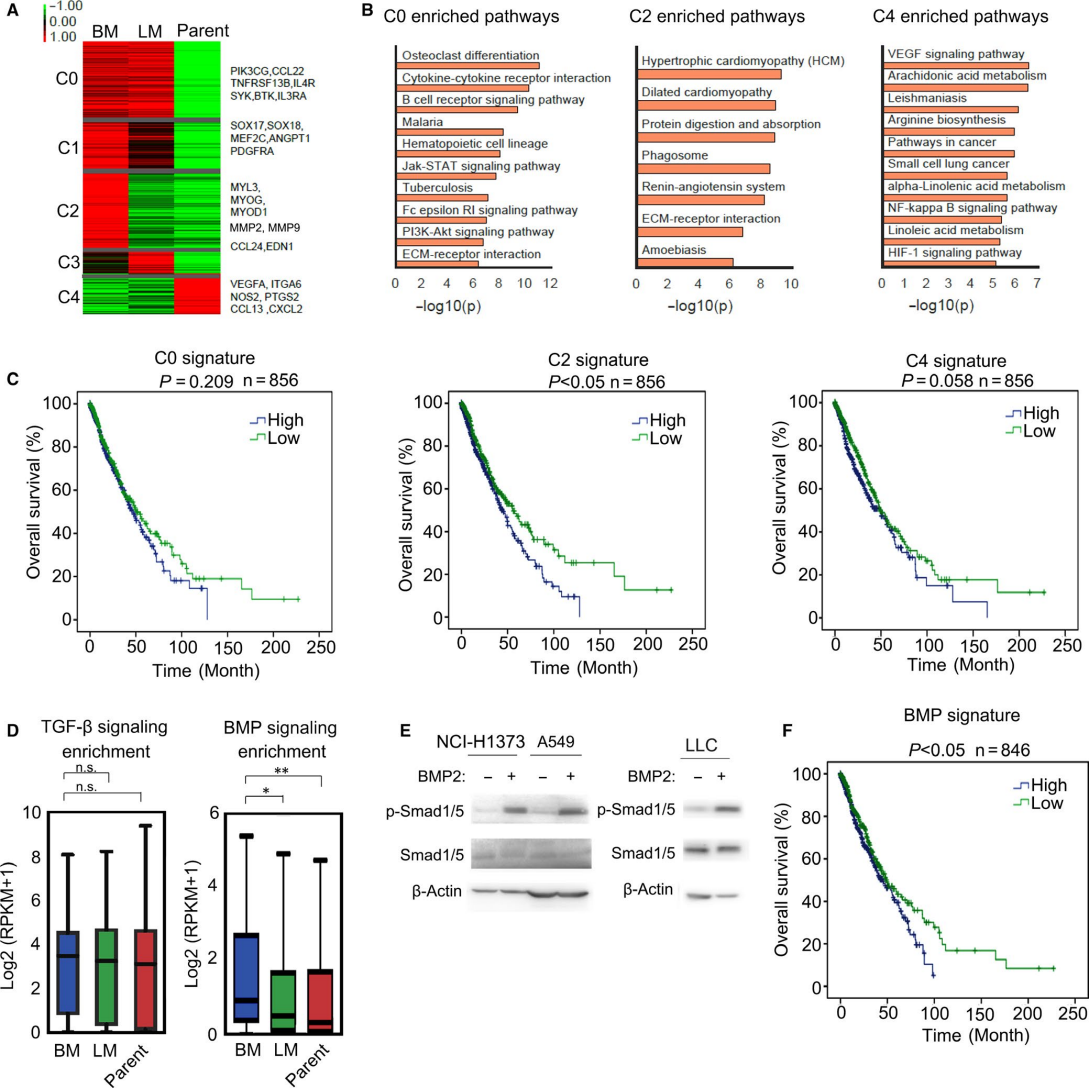
BMP signalling signature expression was up‐regulated in Lewis lung carcinoma bone metastases. A, Heatmap with expression characteristics of the five mRNA‐based clusters in metastatic bone tumours (BM), metastatic lung tumours (LM) and parental Lewis lung cells (Parent). Representative gene lists of each clusters were shown on the right of the heatmap. B, The enriched KEGG pathways of C0, C2 and C4 clusters based on (A). C, The overall survivals of TCGA patients with high C0, C2 or C4 signatures versus low C0, C2 or C4 signatures. The *P value* was based on the log‐rank test. (*P < .05, **P < .01). D, Average expression of TGF‐β or BMP signalling targeted genes in LLCs was defined as TGF‐β or BMP signalling signatures to characterize the activation of TGF‐β or BMP signalling. TGF‐β and BMP signalling signatures in bone metastasis (BM), lung metastasis (LM) and parental Lewis lung cells (Parent) were calculated and shown. The *P value* was based on Student's t test (*P < .05, **P < .01). E, Lysates of NCI‐H1373, A549 and LLC cells were subjected to immunoblotting. F, The overall survival of patients with high BMP signature versus low BMP signature. The *P* value was based on the log‐rank test. (*P < .05, **P < .01)

RNA library preparation was performed as described in the QIAseq Stranded RNA Library Kits (QIAGEN). Total RNA was extracted from mice tissues. The mRNA was fragmented to an average insert size of 200‐400 bp. The cleaved RNA fragments were copied into first‐strand cDNA using reverse transcriptase (Thermo Fisher Scientific) and random primers. The first‐strand cDNA was converted into double‐stranded DNA in the presence of dUTP. These cDNA fragments were subjected to the addition of a single ‘A’ base and subsequent ligation of the adapter. The products were purified and enriched via PCR to generate the final library. After testing quality using Qubit2.0 Fluorometer and Agilent 2100 Bioanalyzer (Agilent Technologies), the libraries were sequenced on the Illumina HiSeq platform (Illumina Inc). Raw sequences were mapped to mouse genome mm10 by STAR (v2.5.4b)^28^ with key parameters setting of ‐‐readMatesLengthsIn Equal ‐‐outFilterMultimapNmax 20 ‐‐alignEndsType Local ‐‐al ignSJDBoverhangMin 10 ‐‐alignMatesGapMax 10 000 ‐‐alignIntronMax 100 000 ‐‐alignSJstitchMismatchNmax 5 −1 5 5 ‐‐outFilterScoreMinOverLread 0.3 ‐‐outFilterMatchNminOverLread 0.3. The expression level FPKM values were obtained from Cuffnorm in Cufflinks package (v2.2.1);^29^ further, significant differential expressed genes (BM vs Parent, LM vs Parent and BM vs LM) were called by Cuffdiff in Cufflinks packages, requiring *P* value < =0.01 and fold change>= 1; all significant differential expressed genes were compiled together to show clusters in Figure 1A. The full dataset can be accessed online at the Gene Expression Omnibus (GEO). The GEO number is GSE148101. The find GO.pl implemented in HOMER^30^ was used to test the enriched GO terms for the target gene lists identified from comparison, with option of human to map mouse genes to human's. Top 10 enriched terms from KEGG pathways were selected to show in figures with requiring of *P* value < 1^e‐5^, and there are fewer than 3000 genes in the term. Expression profiles and clinical data of lung adenocarcinoma (LUAD) and lung squamouse cell carcinoma (LUSC) were downloaded from The Cancer Genome Atlas (TCGA).^31^ The survival analysis was carried based on mean expression for the gene list of different modules.

### 2.18 Statistical analysis

Student's *t* test, one‐way ANOVA test, Wilcoxon rank‐sum test, Fisher's exact test and log‐rank test were used as indicated in the figure legends. *P < *.05 were considered statistically significant.

## 3 RESULTS

### 3.1 BMP signalling signature was up‐regulated in Lewis lung carcinoma bone metastases and predicted poor survival in NSCLC

Lewis lung carcinoma originated from a spontaneous lung adenocarcinoma of a C57BL/6 mouse.^32^, ^33^ Tail vein injection of carcinoma cells was a classical method to establish models of metastasis.^34^, ^35^, ^36^ We injected the Lewis lung carcinoma cells (LLCs) into tail veins of six C57BL/6 mice, resulting in two lung metastases and three bone metastases. RNA‐seq was carried for metastatic bone tumours, metastatic lung tumours and parental Lewis lung cells to analyse the transcriptome differences. The compiled significant differential expressing genes (DEGs) could be classified into five clusters C0, C1, C2, C3 and C4, indicating different expression patterns (Figure 1A). The C0 module showed high expressing level in both metastases, meanwhile C1 and C2 showed higher expressing level in bone metastasis than in lung metastasis and parental cells, and the C3 and C4 module showed the unique highly expressed genes in lung metastasis or parental cells (Figure 1A). Thus, genes in C1 and C2 were more likely to contribute to Lewis lung carcinoma bone metastasis. Consistently, Angiopoietin 1 (ANGPT1) in C1, and MMPs and Endothelin 1 (EDN1) in C2 had been reported to take part in prostate or breast cancer bone metastasis.^23^, ^24^, ^37^, ^38^ Several KEGG^39^ pathways were significantly enriched in C0, C2 and C4 modules but not C1 and C3, as shown in Figure 1B. Enriched KEGG pathways for different modules indicated that Jak‐Stat signalling, and PI3K‐Akt might contribute to lung carcinoma metastasis while VEGF signalling, NF‐κB signalling and HIF‐1α signalling were enriched in parental Lewis lung cells (Figure 1B). Extracellular matrix (ECM)‐receptor interaction and amoebiasis‐associated genes were up‐regulated in the C2 module, indicating that cell deformation and invasion might be significant in the formation of bone metastatic lesions (Figure 1B). Furthermore, we analysed the homologous gene expression features of human based on TCGA data (NSCLC subtypes: LUSC and LUAD)^31^ and found that high expression of bone metastasis‐associated genes in cluster C2 correlated with poor survival in human NSCLC (Figure 1C). What's more, ANGPT1, MMPs and EDN1, majorly up‐regulated in C2, were found as downstream targets of BMP signalling.^40^, ^41^, ^42^ Thus, we speculated that BMP signalling might contribute to Lewis lung carcinoma bone metastasis. Besides, TGF‐β signalling was also important in inducing bone metastasis of various types of cancer, like prostate cancer and breast cancer,^43^ and we went further to analyse the activation of TGF‐β signalling in Lewis lung carcinoma bone metastasis. Average expression of TGF‐β or BMP signalling targeted genes in LLCs were defined as TGF‐β or BMP signalling signatures to characterize the activation of TGF‐β or BMP signalling in bone metastases derived from Lewis lung carcinoma. The TGF‐β target gene list was from KEGG, and BMP target gene list was from our own RNA‐seq results. The RNA‐seq data of LLC with or without BMP2 treatment could be accessed online at the Gene Expression Omnibus (GEO). The GEO number is GSE148101. We found that only BMP signalling signature was up‐regulated in bone metastasis than in lung metastasis and parental Lewis lung cells (Figure 1D). More importantly, up‐regulation of BMP signalling signature genes could predict poor survival in human NSCLC (Figure 1F). In addition, we confirmed that human and mice NSCLC cell lines responded to BMP signalling in vitro (Figure 1E). Taken together, activation of BMP signalling was found in Lewis lung carcinoma bone metastases and correlated with poor survival in NSCLC.

### 3.2 BMP signalling activation was higher in bone metastasis than in lung metastasis of lewis lung carcinoma

**Figure 2:**
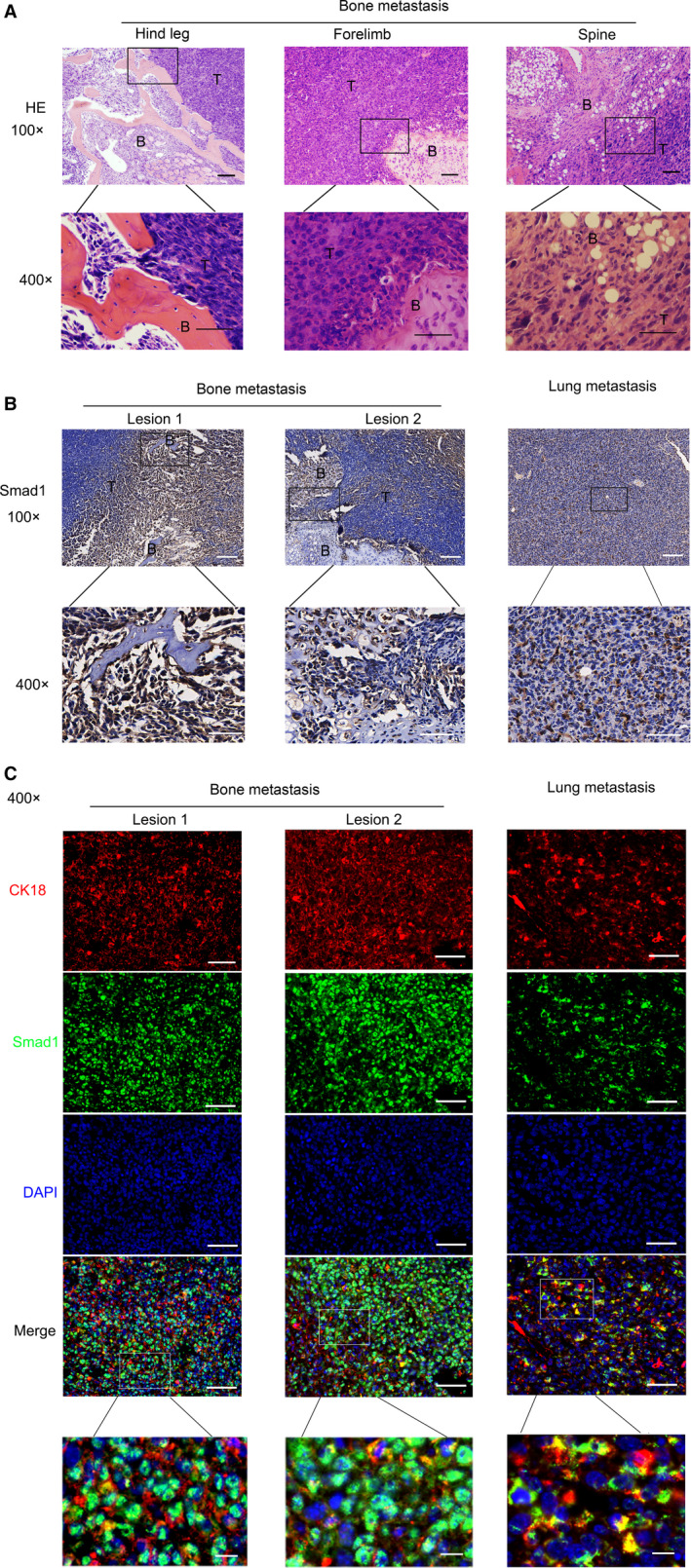
BMP activation was higher in bone metastasis than in lung metastasis of Lewis lung carcinoma. A, Lewis lung carcinoma cells (LLCs) were injected into tail veins of C57/BL6 mice, resulting in lung metastasis and bone metastasis. Representative HE staining of metastatic bone tumours from limbs or spines was shown. Scale bars, 100 μM. Regions in the rectangles were magnified to 400×. Scale bars of the 400 × photos were 50 μM. T: Tumour; B: Bone. B, Representative Smad1 immunohistochemical staining of metastatic bone tumours and metastatic lung tumours metastasis tissues. Scale bars of the 100 × photographs were 100μM. Regions in the rectangles were magnified to 400×. Scale bars of the 400 × photographs were 50μM. T: Tumour; B: Bone. C, Representative immunofluorescence staining of Smad1, CK18 and DAPI. Scale bars of the 400 × photos were 50 μM. Regions in the rectangles were magnified, scale bars: 10 μM. Red: CK18‐TRITC; Green: Smad1‐FITC; Blue: DAPI

We found that LLCs could metastasize to both limbs and spines to form metastatic lesions (Figure 2A). When BMP signalling is activated, Smad1/5/8 is phosphorylated and translocated into nucleus with Smad4.^44^, ^45^ In our results, we found that Smad1 was expressed in the nucleus of Lewis lung carcinoma cells at the invasive sites, where bone resorption and destruction occurred (Figure 2B‐C). Those results suggested that BMP signalling was activated in metastatic bone tumours derived from Lewis lung carcinoma. CK18, as a kind of cytokeratin, frequently expressed in NSCLC.^46^, ^47^, ^48^ We stained Lewis lung carcinoma cells with CK18 and Smad1 to further confirm that BMP signalling was activated in Lewis lung carcinoma cells rather than other cell types of metastatic bone tumours. As shown in Figure 2C, nuclear Smad1 staining was usually found in CK18^+^ LLCs in metastatic bone tumours, indicating that BMP signalling of Lewis lung carcinoma cells were activated in bone metastases.

Smad1 was expressed both in lung metastasis and bone metastasis. However, the location of Smad1 within the two metastatic tissues was different (Figure 2B‐C). Smad1 majorly located in the nucleus at the invasive boundaries in bone metastasis, while it diffused in both the cytoplasm and nucleus of carcinoma cells in lung metastasis (Figure 2B‐C, Figure S1A‐B). Moreover, higher phosphorylated Smad1/5 was found in bone metastasis in comparison with lung metastasis (Figure S1C‐D). Therefore, the activation of BMP signalling was higher in bone metastasis than in lung metastasis of Lewis lung carcinoma. Our results indicated that BMP signalling may probably play a more important role in bone metastasis than in lung metastasis of NSCLC.

### 3.3 BMP2 signalling activation enhanced bone metastasis of lewis lung carcinoma in vivo

**Figure 3:**
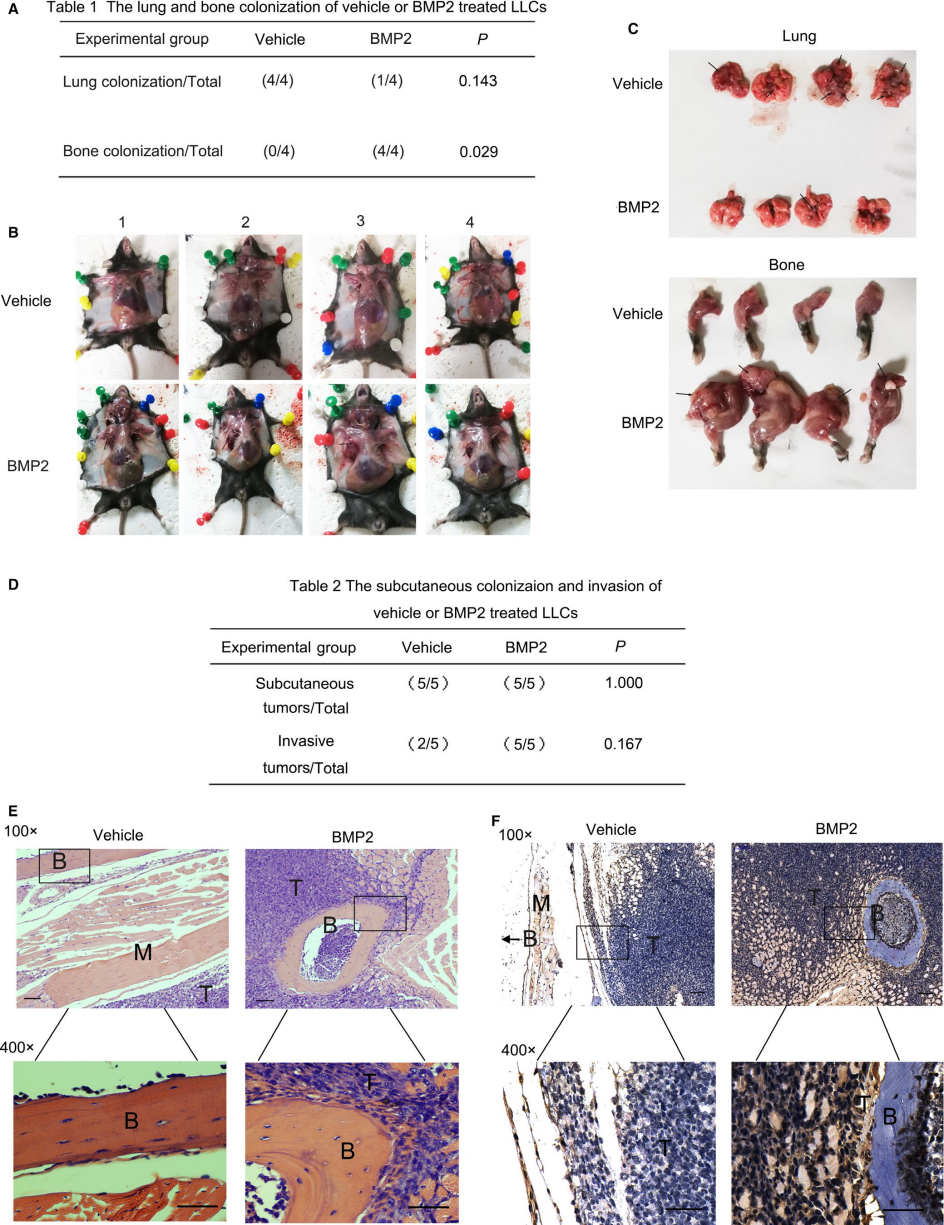
BMP2 signalling activation enhanced bone metastasis of Lewis Lung Carcinoma in vivo. A, 1ⅹ10^6^ LLCs with vehicle or BMP2 treatment were injected into the left lung lobes of C57BL/6 mice. The number of lung or bone tumour‐burdened mice and total mice (n = 4) was recorded. The *P* values were based on Fisher's exact test. B, Graphs of tumour‐burdened mice in (A). Black arrows showed the bone metastatic lesions. C, Graphs of lungs and forelimbs harvested from mice in (A). Black arrows showed the metastatic lesions. D, 1 × 10^5^ the vehicle or 20 ng/mL BMP2 pre‐treated LLCs were injected into the left hind legs of C57BL/6 mice subcutaneously. To retain the BMP2 signalling activation in the hind leg muscles, 3 μg/kg vehicle or BMP2 was further injected into the left hind leg subcutaneously per week. The number of subcutaneous or invasive tumour‐burdened mice and total mice (n = 5) was recorded. The *P* values were based on Fisher's exact test. E, Representative HE staining of tissues from hind legs in (E). Scale bars of the 100 × photos were 100 μM. Regions in the rectangles were magnified to 400×. Scale bars of the 400 × photographs were 50 μM. T: Tumour; B: Bone; M: Muscle. F, Representative Smad1 immunohistochemical staining of tissues derived from hind legs in (E). Scale bars of the 100 × photos were 100 μM. Regions in the rectangles were magnified to 400×. Scale bars of the 400 × photographs were 50 μM. T: Tumour; B: Bone; M: Muscle. In the vehicle group, the arrow indicated that the bone was outside the field of view

BMP signalling was found activated in bone metastasis of Lewis lung carcinoma according to the results above, and the high expression of BMP2 has been reported in NSCLC.^6^, ^7^, ^8^, ^9^, ^10^ Thus, we focused on the direct roles of BMP2 in bone metastasis of NSCLC. We pre‐treated LLCs with vehicle or 20 ng/mL BMP2 for 24h. After that, we injected the vehicle or 20 ng/mL BMP2 pre‐treated LLCs into the left lung lobes of C57BL/6 mice. We found that BMP2‐treated LLCs were more likely to colonize in left ribs and shoulders while vehicle treated LLCs were more likely to colonize in lungs (Figure 3A‐C).

We went further to investigate the roles of BMP signalling playing in the bone invasion of NSCLC cells in vivo. We pre‐treated LLCs with vehicle or 20 ng/mL BMP2 for 24 hours. After that, we injected the vehicle or 20 ng/mL BMP2 pre‐treated LLCs into the left hind legs of C57BL/6 mice subcutaneously to analyse the direct invasion of Lewis lung carcinoma in the hind legs. To retain the BMP2 signalling activation in the hind leg muscles, we repetitively injected 3 μg/kg vehicle or BMP2 into the left hind leg subcutaneously per week for three weeks. In the vehicle group, we found that the carcinomas majorly colonized subcutaneously and grew separately from the muscles and bones, indicating that Lewis lung carcinoma did not invade into muscles and bones (Figure 3D‐F). Differently, in the BMP2 group, the muscles had been destructed and the carcinoma had invaded into the bones (Figure 3E‐F). Although the difference was not statistically significant in numbers which could be attribute to the small number of mice investigated, BMP2 still had the trend to enhance the migration and invasion of Lewis lung carcinoma cells in vivo. The activation of BMP2 signalling was confirmed by nuclear Smad1 staining and the expression of phosphorylated Smad1/5 (Figure 3F, Figure S2A‐C).

### 3.4 BMP2 signalling enhanced migration and invasion of NSCLC cells

**Figure 4:**
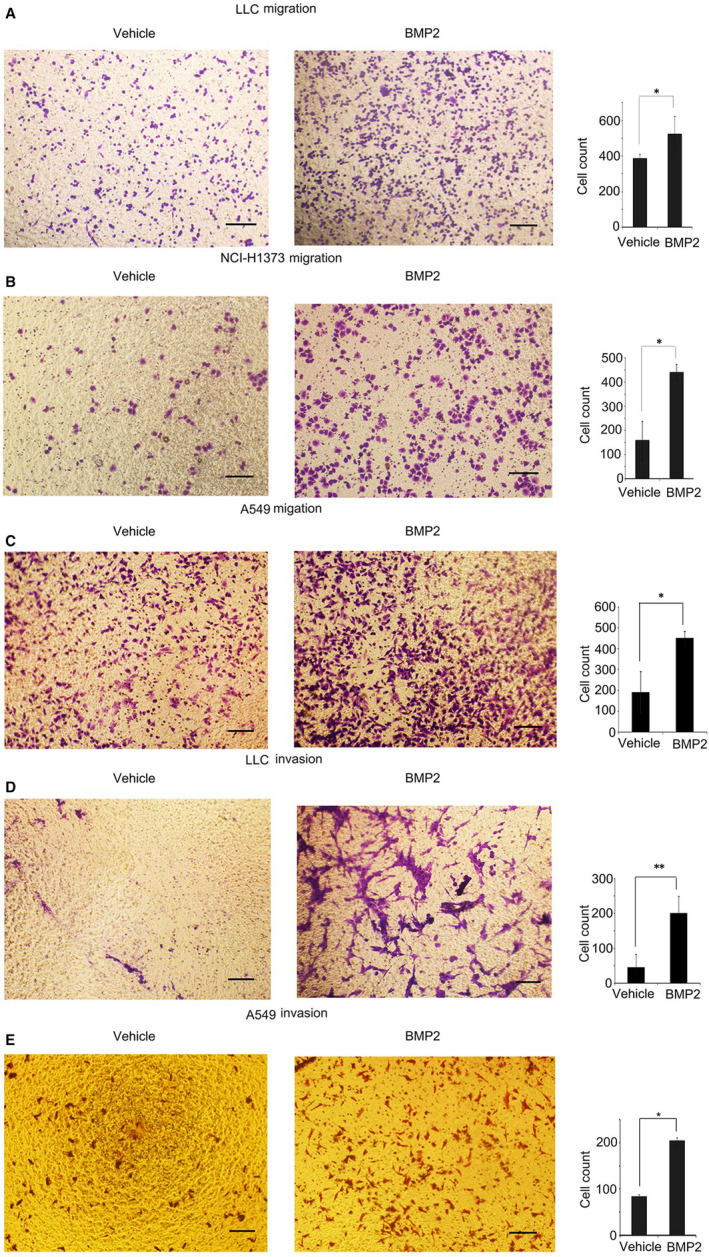
BMP2 signalling enhanced migration and invasion of NSCLC cells. A‐C, 1 × 10^4^ LLCs, NCI‐H1373 cells and A549 cells in culture media without FBS were placed on the upper layer of cell culture insert with polycarbonate membrane (Transwell^@^, 8.0 μm pore size, Corning). The complete culture media with or without 20 ng/mL BMP2 were placed below the cell permeable membrane. The cells migrating to the bottom of the membrane were stained with crystal violet and counted. Representative photographs were shown. Scale bars, 100 μM. Average cell numbers of at least three fields were shown on the right. The *P value* was based on Student's t test. (*P < .05, **P < .01). D‐E, Corning cell culture insert with polycarbonate membrane (Transwell^@^, 8.0μm pore size, Corning) were pre‐treated with 10:1 DMEM and matrigels (BD BioSciences). 1 × 10^5^ LLCs and A549 cells were treated the same way as LLCs in (A). The cells invading to the bottom were stained with crystal violet and counted. Representative photos were shown. Scale bars, 100 μM. Average cell numbers of at least three fields were shown on the right. The *P value* was based on Student's t test. (*P < .05, **P < .01)

According to our transcriptome results, ECM‐receptor interaction and amoebiasis‐associated genes were up‐regulated in bone metastasis of Lewis lung carcinoma, indicating that cell deformation, migration and invasion might play significant roles in the formation of bone metastasis (Figure 1B). And BMP2 could enhance the migration and invasion of Lewis lung carcinoma in vivo (Figure 3F). Therefore, we examined whether BMP2 signalling could enhance migration and invasion of NSCLC cells in vitro via the transwell assay. We found that BMP2 could enhance the migration of NSCLC cells, like LLCs, NCI‐H1373 cells and A549 cells in vitro (Figure 4A‐C). What's more, the invasive ability of LLCs and A549 cells was also promoted by BMP2 (Figure 4D‐E).

### 3.5 Stroma fibroblasts would be the nature source to secret BMP2 in bone metastasis of lewis lung carcinoma

**Figure 5:**
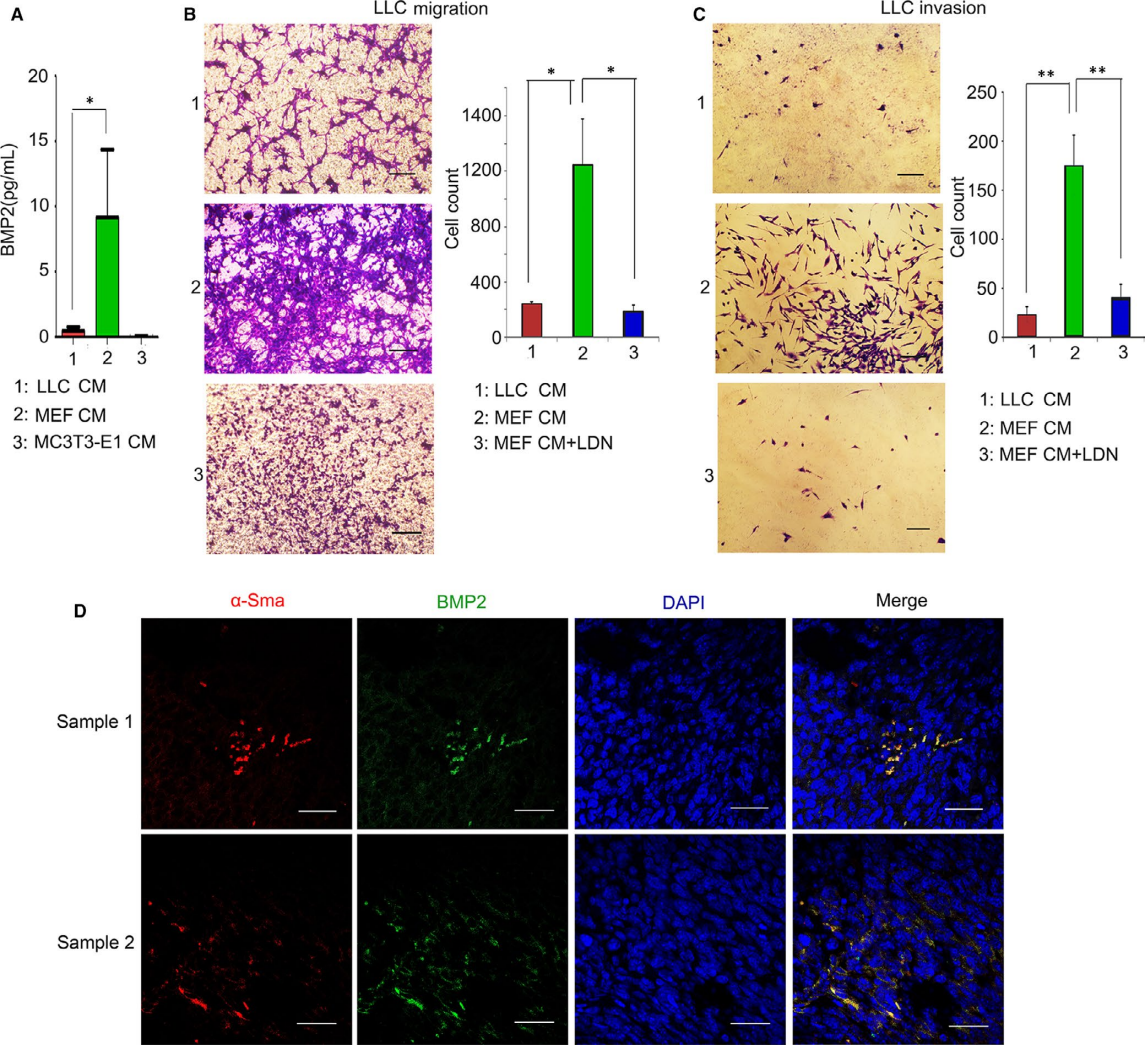
Stroma fibroblasts would secret BMP2 in bone metastasis of Lewis lung carcinoma. A, 1 × 10^4^ LLCs, MEF or MC3T3‐E1 cells were cultured with the same volume of DMEM with 10% FBS for 3 days. The concentration of BMP2 in the supernatant were measured by ELISA. The *P value* was based on one‐way ANOVA test. (*P < .05, **P < .01). B, 1 × 10^4^ LLCs in culture media without FBS were placed on the upper layer of Corning cell culture insert with polycarbonate membrane (Transwell^@^, 8.0 μm pore size, Corning). Conditional media from 1 × 10^4^ LLC or MEF cells were placed below the cell permeable membrane with the indicated treatment. The cells migrating to the bottom were stained with crystal violet and counted. Representative photographs were shown. Scale bars, 100 μM. Average cell numbers of at least three fields were shown on the right. The *P value* was based on Student's *t* test. (*P < .05, **P < .01). C, Corning cell culture insert with polycarbonate membrane (Transwell^@^, 8.0 μm pore size, Corning) were pre‐treated with 10:1 DMEM and matrigels (BD BioSciences). 1 × 10^5^ LLCs in culture media without FBS were placed on the upper layer of the diluted matrigels. Conditional media from 1 × 10^4^ LLC or MEF cells were placed below the cell permeable membrane with the indicated treatment. The cells invading to the bottom were stained with crystal violet and counted. Representative photographs were shown. Scale bars, 100 μM. Average cell numbers of at least three fields were shown on the right. The *P value* was based on Student's *t* test. (*P < .05, **P < .01). D, Representative immunofluorescence photographs of BMP2, α‐Sma and DAPI in metastatic bone tumours of Lewis lung carcinoma. Scale bars of the 400 × photographs were 50 μM. Red: α‐Sma‐TRITC; Green: BMP2‐FITC; Blue: DAPI

As BMP2 signalling played an important role in bone metastases, exploring the nature source of BMP2 in bone metastasis was an important issue that needed to be validated. Rajski et al had reported that high BMP2 derived from stroma cells correlated with poor outcome in lung adenocarcinoma.^13^ Tumour stroma fibroblast cells secreted diverse cytokines to enhance cancer progression.^49^, ^50^ In our result, we found that MEF (mice embryonic fibroblast) could secrete much more BMP2 than LLCs and the pre‐osteoblast cells MC3T3‐E1(Figure 5A). Moreover, the conditional media of MEF could promote the migration of LLCs (Figure 5B). Furthermore, in consistent with the migration phenomena, the conditional media of MEF could enhance the invasion of LLCs (Figure 5C). Interestingly, we found that the bone morphogenetic protein receptor type I (BMPRI) inhibitor LDN‐193189 could inhibit the migration and invasion of LLCs induced by MEF conditional media (CM) (Figure 5B‐C), indicating that MEF enhance migration of LLCs via secreting BMP2. As numerous cell types were growing within the metastatic bone tumours, we investigated the expression of BMP2 in bone metastasis through immunofluorescence assay. We also found that α‐Sma^+^ marked fibroblasts expressed more BMP2 than other cell types in bone metastases of Lewis lung carcinoma (Figure 5D). The results above indicated that fibroblast cells might be the major source of BMP2. BMP2 originated from stroma fibroblasts contributed to the migration and invasion of Lewis lung carcinoma cells.

### 3.6 BMP2, in combination with NSCLC cells and osteoblast cells, regulated the osteolytic and osteoblastic mechanisms of bone metastasis

In osteolytic metastases, the destruction of bone was mediated by osteoclasts rather than tumour cells.^51^, ^52^ BMP2 alone generally could not induce osteoclast‐genesis, but it had also been reported to stimulate the activation of osteoclasts in combination with titanium particles or chondrocytes through inducing the expression of RANKL.^53^, ^54^ Osteoblasts and NSCLC cells in tumour microenvironment could cooperate with each other to induce macrophages differentiation into osteoclasts.^51^ Thus, we continued to examine whether BMP2 could contribute to this process with NSCLC and osteoblast cells synergistically. We co‐cultured the macrophage cell line RAW264.7 with LLC and MC3T3‐E1 or A549 and MC3T3‐E1 cells to induce its osteoclasts differentiation for 7 days. We found that without BMP2 stimulation, few macrophages could differentiate into TRAP^+^ osteoclast cells (Figure S3A‐B). However, BMP2 could strongly enhance the differentiation of macrophages into osteoclasts under the co‐culture condition, indicating that BMP2 signalling contributed to osteolytic metastasis (Figure S3A‐B). The bi‐directional interactions between tumour cells and osteoclasts led to both osteolysis and tumour growth.^51^, ^55^

**Figure 6:**
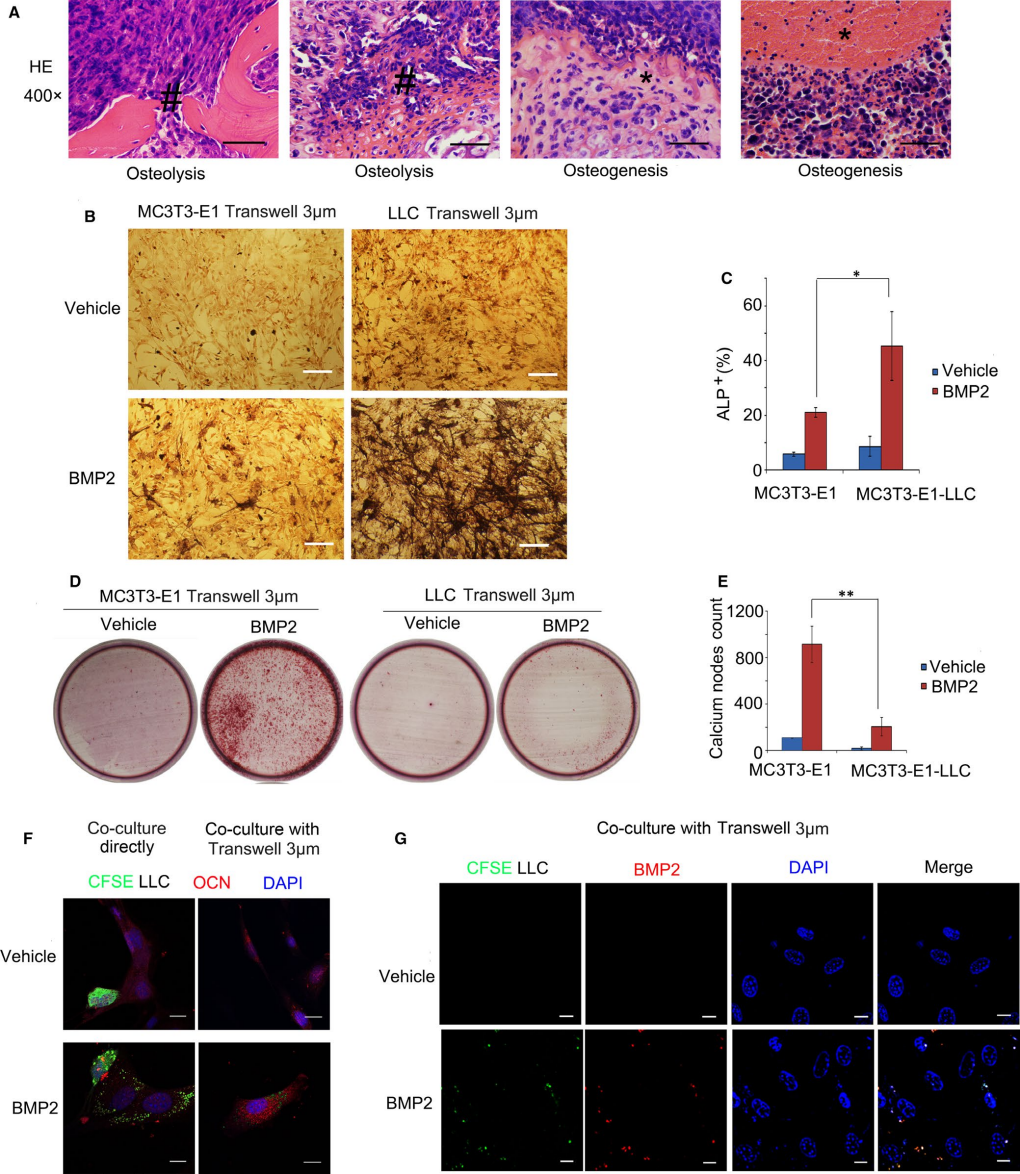
BMP2 regulated the osteolytic and osteoblastic mechanisms of bone metastasis in Lewis lung carcinoma. A, 1 × 10^6^ LLCs were injected into tail veins of C57BL/6 mice, resulting in bone metastasis. Representative HE staining of bone metastasis tissues from limbs or spines was shown. Lysed bones (#). Immature bones (*). Scale bars, 50 μM. B, LLCs or MC3T3‐E1 cells were placed on the upper layer of cell culture insert with polycarbonate membrane (Transwell^@^, 3.0μm pore size, Corning). MC3T3‐E1 cells were cultured below in culture media with or without 200ng/mL BMP2 for seven days. ALP staining for MC3T3‐E1 cells cultured below was conducted. Representative photographs were shown. Scale bars, 10μM. C, Average ALP^+^ cell numbers of at least three fields in (C) were shown. The *P value* was based on Student's t test. (*P < .05, **P < .01). D, LLCs or MC3T3‐E1 cells were treated the same way as shown in (B) for 14 days. MC3T3‐E1 cells cultured below were stained with 1% Alizarin red (pH = 4.2). Representative photos were shown. E, Average calcium node numbers in (F) of at least three wells were shown. The *P value* was based on Student's t test. (*P < .05, **P < .01). F, 3 × 10^4^ MC3T3‐E1 cells were co‐cultured with 3 × 10^4^ CFSE‐labelled (green) LLCs directly (left). 3 × 10^4^ MC3T3‐E1 cells were seeded into the wells of the 6‐well co‐culture plates (Corning), and 3 × 10^4^ CFSE‐labelled (green) LLCs were seeded on the upper layer of Corning cell culture insert with polycarbonate membrane (Transwell^@^, 3.0 μm pore size, Corning) with 200 ng/mL BMP2 or vehicle (right). OCN‐TRITC (red) was stained to show MC3T3‐E1 cells. DAPI (blue) was stained to show the nucleus. Representative immunofluorescence photographs were shown. Scale bars, 10 μM. G, 3 × 10^4^ MC3T3‐E1 cells and 3 × 10^4^ CFSE‐labelled (green) LLCs were seeded on the Corning transwell the same way as shown in (F). BMP2 were stained (Red). DAPI (blue) was stained to show the nucleus. Representative immunofluorescence photographs were shown. Scale bars, 10 μM

However, in bone metastasis of Lewis lung carcinoma, we could see both the destruction of mature bone tissues and the formation of immature bone tissues, indicating that osteoblastic mechanisms might also play roles in bone metastases of Lewis lung carcinoma (Figure 6A). Although BMP2 was a well‐known protein with bone inductivity, how BMP2 in combination with NSCLC cells to regulate the osteoblastic mechanism in bone metastases of Lewis lung carcinoma was largely unknown. We co‐cultured LLCs in the Corning cell culture insert with polycarbonate membranes (Transwell@, 3.0μm pore size, Corning) with BMP2 and pre‐osteoblasts MC3T3‐E1 below in the plates to mimic the neoplastic osteogenesis in vivo. In the co‐culture system, LLCs could not contact MC3T3‐E1 cells directly but the vesicles and cytokines secreted by LLCs could contact MC3T3‐E1 cells through the 3μm pores. We found that BMP2 could induce ALP^+^ differentiated MC3T3‐E1 cells, while LLCs could further enhance this process (Figure 6B‐C). However, the formation of calcium nodes induced by BMP2, which indicated the mature osteocytes, was inhibited by LLCs (Figure 6D‐E). In addition, we found that LLCs alone could enhance the expression of the proliferation marker Ki67 in MC3T3‐E1 cells and attenuate MC3T3‐E1 apoptosis independent of BMP2, which might be another mechanism supporting neoplastic osteogenesis (Figure S4A‐B). The results above revealed that BMP2 induced immature osteoblasts differentiation with NSCLC cells synergistically.

We went further to study how LLCs enhanced BMP2 to induced osteoblasts differentiation. We co‐cultured LLCs with MC3T3‐E1 cells directly. LLCs were stained with CFSE (green), and MC3T3‐E1 cells were stained with the anti‐OCN antibody (red, TRITC‐conjugated secondary antibody labelled). Interestingly, under BMP2 treatment, LLCs could secrete substances (green stained), which subsequently attached to MC3T3‐E1 cells (red stained) (Figure 6F). Moreover, we indirectly co‐cultured LLCs in the Corning cell culture insert with polycarbonate membranes (Transwell@, 3.0 μm pore size, Corning) and found that the substance (green stained) that secreted by LLCs could pass through the 3.0 μm pores and attached to the MC3T3‐E1 cells below (Figure 6F). Furthermore, we found that BMP2 co‐localized with the substance secreted by LLCs (Figure 6G). Taken together, the results above indicated that LLCs enriched BMP2 to pre‐osteoblast cells to induce the early osteoblast differentiation, which might be a potential mechanism about how BMP2 induced immature osteogenesis with LLCs synergistically.

## 4 DISCUSSION

NSCLC is the third most common bone metastasis carcinoma, following breast cancer and prostate cancer.^56^, ^57^ However, different from breast cancer and prostate cancer, researches focused on mechanisms of NSCLC bone metastases are rare. Therefore, new mechanisms about NSCLC bone metastases and new targets for NSCLC bone metastasis therapy need to be researched. In our present work, we find that BMP2 signalling plays roles in NSCLC bone metastasis.

**Figure 7:**
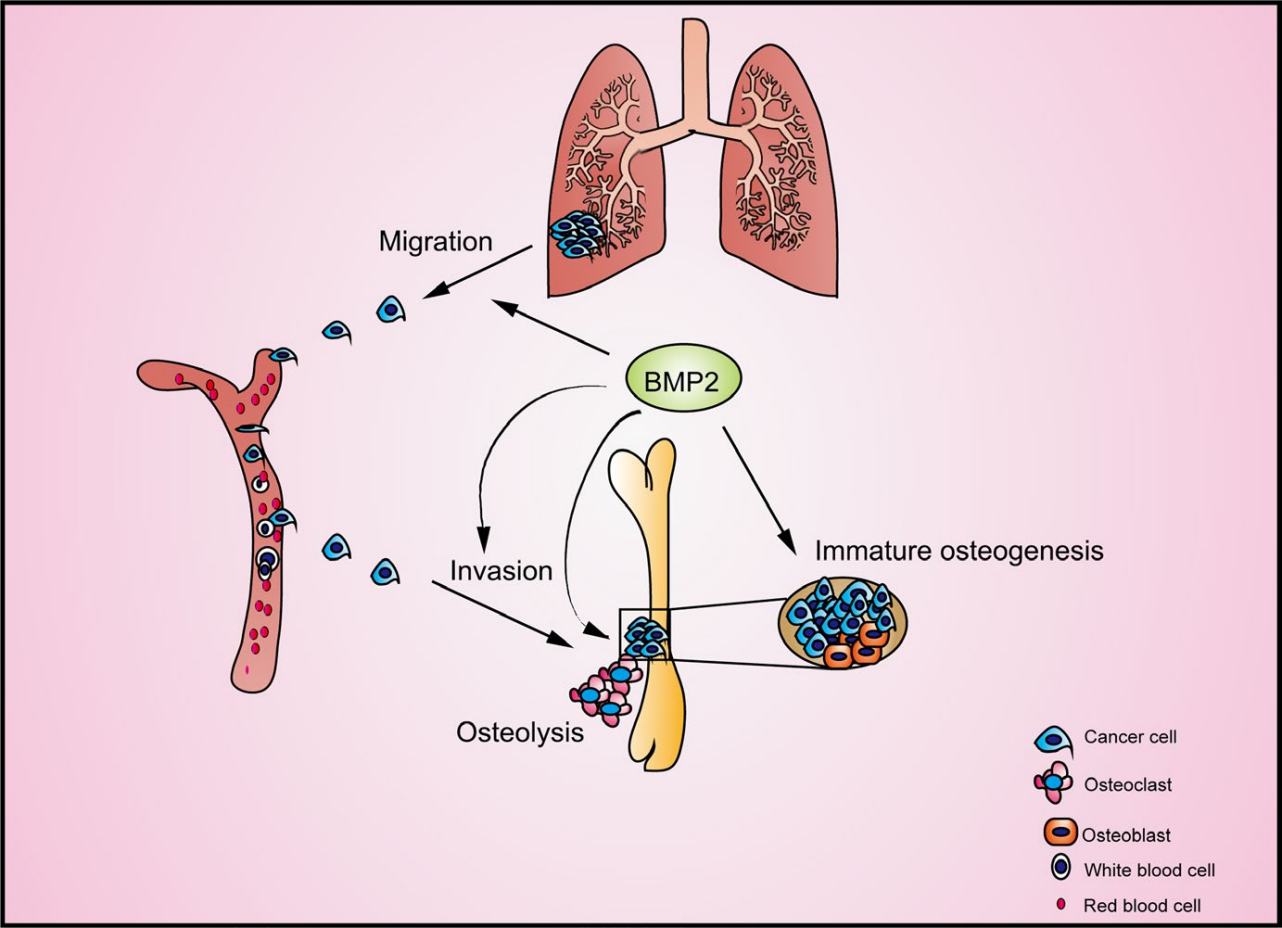
The schematic diagram illustrated roles of BMP2 signalling plays in enhancing bone metastases of NSCLC

Formation of bone metastatic lesions needs collaboration of lung cancer cells and the microenvironment.^18^, ^55^ We find that BMP2 signalling can promote bone metastases of lung carcinoma from multiple directions (Figure 7). At the early stage, BMP2 enhances lung cancer cells to migrate and invade to the bone tissues. BMP2 signalling regulates the interaction among lung cancer cells, osteoblasts and macrophages to promote the osteoclasts differentiation, further enhancing osteolysis in bone metastases. BMP2 signalling can also play roles in the osteoblast differentiation to induce the immature bone formation within the bone metastases. Nevertheless, the osteoblasts cannot form mature bone tissues. It has been reported that cancer cells rely on direct calcium influx from osteogenic cells to form bone metastatic lesions.^58^, ^59^ Thus, the immature bone tissues induced by BMP2 signalling may be the source of cytokines or matrix for the metastatic lesions maintaining.

The osteoclastic bone resorption is significant to make the skeleton a permissive environment in most bone metastases.^60^ Osteoclasts, the only cells capable of bone resorption, derived from myeloid lineage, were reported to be controlled by RANKL, PTHrP and Jagged 1.^61^, ^62^, ^63^, ^64^ In a recent report, LIGHT (tumour necrosis factor superfamily member 14, TNFSF14), derived from monocytes, was shown to promote osteolysis in NSCLC bone metastases and could be a new therapy target.^65^ RANKL can be secreted by osteoblast lineages or stromal cells, and PTHrp can be derived from tumour cells.^61^, ^66^, ^67^, ^68^ Although PTHrP and RANKL have been reported to be associated with NSCLC bone metastasis,^69^, ^70^ therapy efficiency of treatment targeting RANKL on NSCLC bone metastases is extremely limited. Thus, mechanisms about how RANKL is expressed and secreted need to be researched.^67^ In our study, BMP2 is found to promote osteoclast differentiation of macrophages in cooperation with pre‐osteoblasts and NSCLC cells, indicating its roles in osteolytic mechanism of NSCLC bone metastases. It has been reported that BMP2 properly enhances osteoclast activation indirectly through inducing secretion of RANKL.^53^, ^54^, ^67^ It needs to be further researched whether this mechanism works in NSCLC‐associated osteolysis.

Osteoblastic mechanism of bone metastasis remains much more unexplored than osteolysis mechanism. We firstly report the osteoblastic mechanism of NSCLC bone metastasis and find that BMP2 signalling plays significant roles in the formation of immature bone tissues within NSCLC bone metastatic lesions. Membrane‐associated microvesicles from stromal cells or other cells in the osteogenesis niche are important carriers for BMP2 delivered to mesenchymal stem cells or pre‐osteoblasts.^71^ In prostate cancer bone metastasis, cancer cells release BMP2 via microvesicles to promote pre‐osteoblasts differentiation.^72^ Interestingly, according to our results, fibroblasts are the origination of BMP2 rather than NSCLC cells and osteoblasts. However, NSCLC can collect BMP2 from the environment and deliver to pre‐osteoblast cells via a substance to enhance the osteoblasts differentiation, which may be a new mechanism about how NSCLC cells interact with pre‐osteoblast cells. Furthermore, it needs further research whether the substances with BMP2 secreted by LLCs are microvesicles.

BMPs belong to TGF‐β superfamily, including BMP2, BMP4, BMP6, BMP7 and so on.^73^ The roles of other BMP family members in lung cancer have also been reported. Different from BMP2, BMP6 has been reported to be a tumour suppressor in lung cancer, which may be epigenetically silenced in lung cancer.^74^ The roles of BMP4 and BMP7 in lung cancer may be controversial. Some researches demonstrate that BMP4 and BMP7 enhance invasion and metastasis of lung cancer while others do not, which may need to be further confirmed.^75^, ^76^, ^77^

In the future, different inhibitors targeting BMP signalling factors can be developed for the therapy of NSCLC bone metastasis. As various kinase inhibitors have been developed, BMP type I receptors ALK2/3/6, and BMP type II receptors BMPRII, ActRII and ActRIIB, can be good targets.^78^ Besides, BMP2 antagonisms or antibodies targeting BMP2 can also be tried in the treatment of NSCLC bone metastasis.

## 5 CONCLUSIONS

BMP2 signalling is activated in bone metastases of NSCLC. Moreover, BMP2 signalling activation correlates with poor survival in human NSCLC. Furthermore, BMP2, majorly derived from stroma fibroblasts, enhances bone metastases of NSCLC not only through promoting migration and invasion of cancer cells, but also through both osteolytic and osteoblastic mechanisms. Altogether, inhibition of BMP2 signalling can be a potential therapy choice for preventing bone metastases of NSCLC patients.

## CONFLICT OF INTERESTS

The authors declare that they have no competing interests.

## AUTHORS' CONTRIBUTIONS

Fei Huang: Conceptualization; data curation; formal analysis; funding acquisition; roles/writing – original draft. Yaqiang Cao: Conceptualization; data curation; formal analysis; writing – review and editing. Gui Wu: Data curation; formal analysis; writing – review and editing. CaihongWang: Data curation. Junying Chen: Data curation. Wanzun Lin: Data curation. Ruilong Lan: Investigation. Bing Wu: Investigation. Xianhe Xie: Investigation. Jinsheng Hong: Investigation. Lengxi Fu: Investigation.

## Supporting information

Fig S1‐S4

## Data Availability

The data that support the findings of this study are available from the corresponding author.
