# Supplementary material for: BMP2 signalling activation enhances bone metastases of non‐small cell lung cancer
Source: J Cell Mol Med. 2020 Aug 4;24(18):10768–84. doi: 10.1111/jcmm.15702 (PMC7521321; doi:10.1111/jcmm.15702)
Supplement: Supplementary file 1 — Fig S1‐S4 [file JCMM-24-10768-s001.docx]

**Supplementary**

**
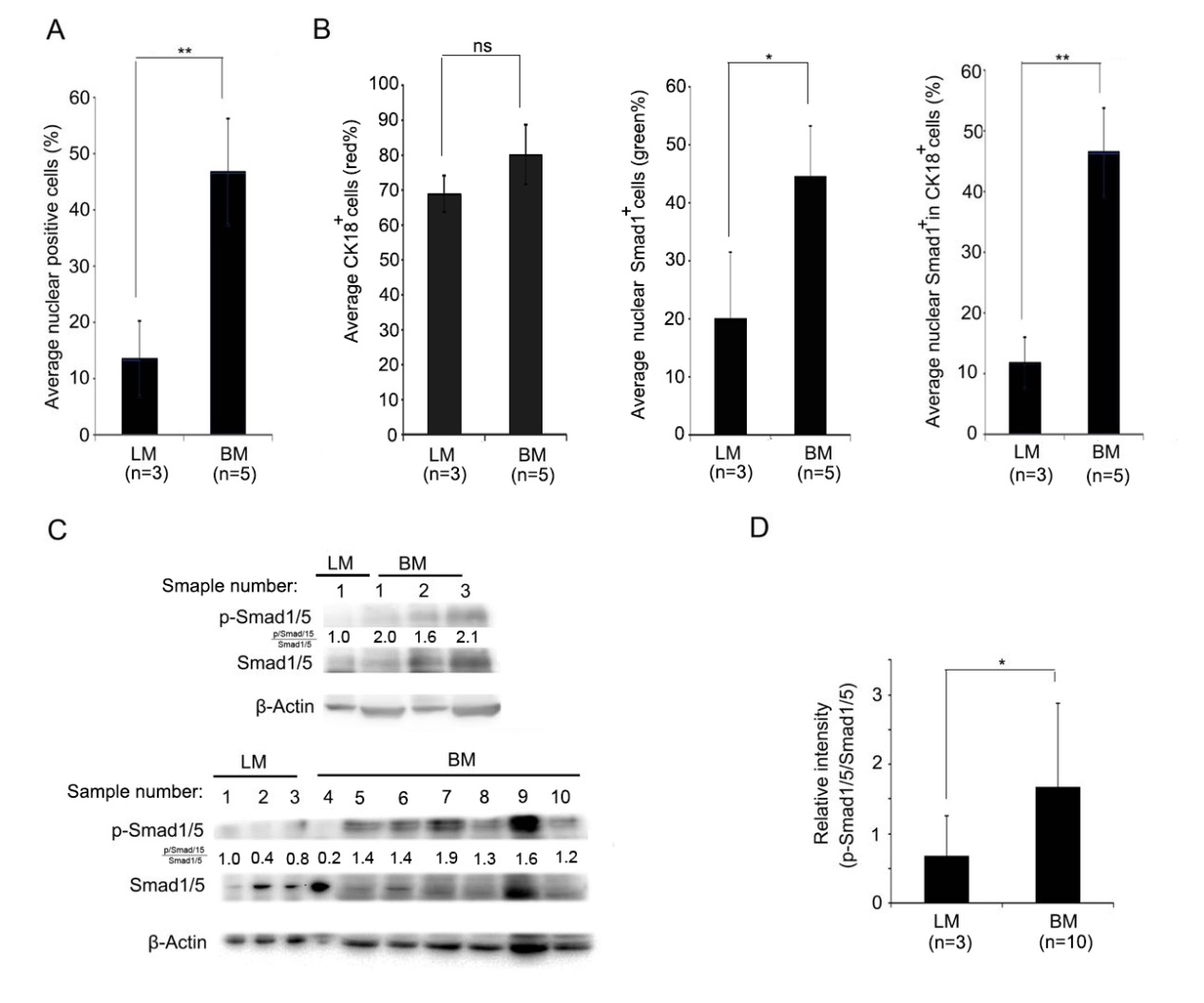
**

**Fig Sup 1. BMP signaling is activated in metastatic bone tumors of Lewis lung carcinoma.**

1. Average percentages of nuclear Smad1 positive cells of at least three samples were shown. Representative immunohistochemical images were shown in Fig 2(B). LM: metastatic lung tumors (n=3), BM: metastatic bone tumors (n=5). The *p value* was based on the student’s t-test. (**P<0.5, **: P<0.01*).
2. Average percentages of CK18 positive cells (red), nuclear Smad1 positive cells (green) in all cells (blue) and nuclear Smad1 positive cells (green) in CD18 positive cells (red) of at least three samples were shown. Representative immunofluorescence images were shown in Fig 2(C). LM: metastatic lung tumors (n=3), BM: metastatic bone tumors (n=5). The *p value* was based on the student’s t-test. (**P<0.5, **: P<0.01*).
3. Lysates of metastatic lung tumors (LM, n=3) or metastatic bone tumors (BM, n=10) were indicated to immunoblotting. The band intensity of was normalized to β-Actin. The ratio of p-Smad1/5 and Smad1/5 bands intensity was analyzed and shown.
4. The average relative band intensity of the experiments in (C) were calculated and shown. LM: metastatic lung tumors (n=3), BM: metastatic bone tumors (n=10). The *p value* was based on the student’s t-test. (**P<0.05*)


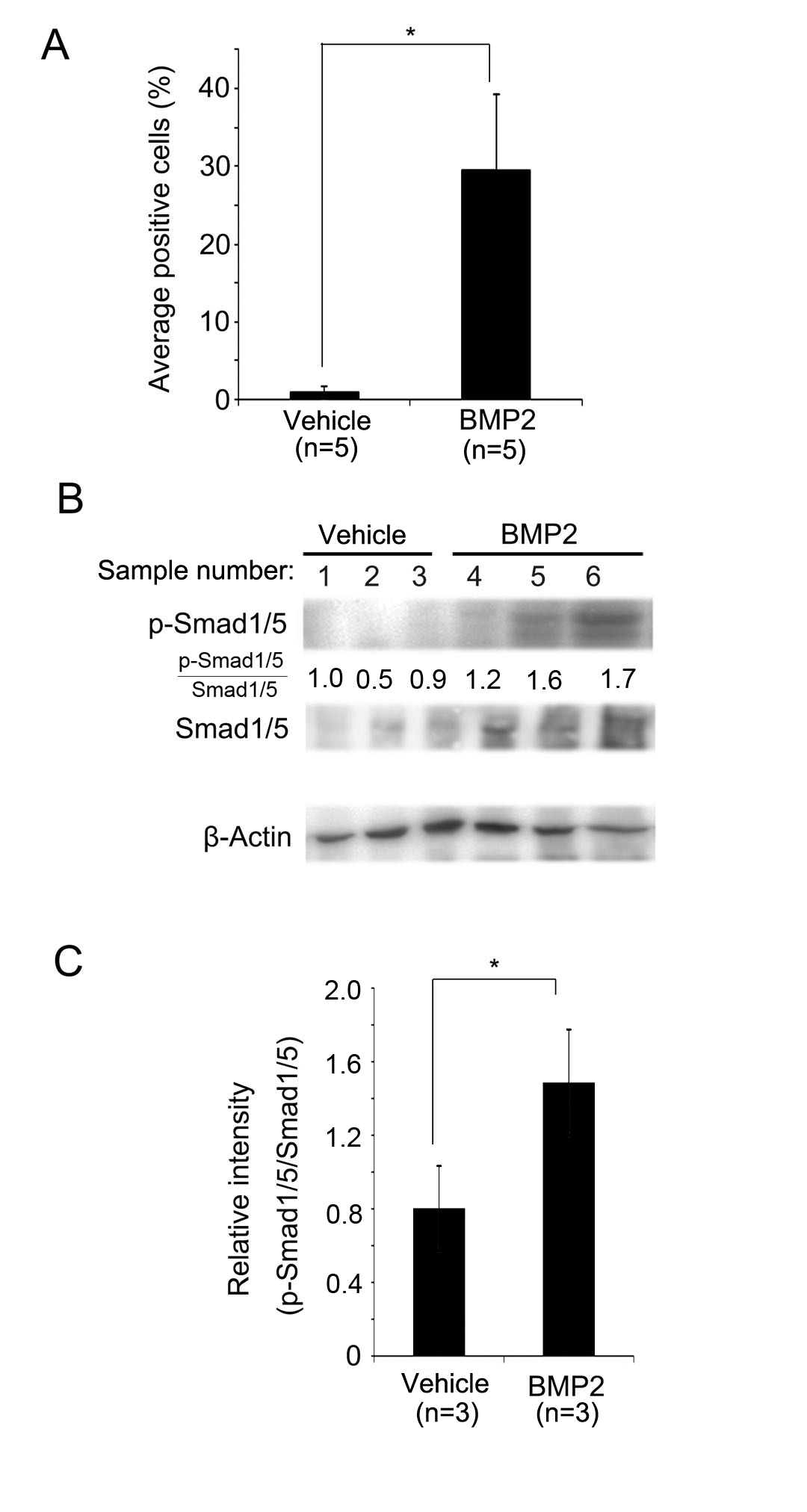


**Fig Sup 2. BMP2 signaling was activated in tumors formed by BMP2 pre-treated LLCs.**

1. Average percentages of nuclear Smad1 positive cells of at least three samples were shown. Representative immunohistochemical images were shown in Fig 3G. Vehicle group: n=5, BMP2 group: n=5. The *p value* was based on the student’s t-test. (**P<0.5, **: P<0.01*).
2. Lysates of tissues derived from hind legs of vehicle treated mice (n=3) or BMP2 treated mice (n=3) were indicated to immunoblotting. The band intensity of was normalized to β-Actin. The ratio of p-Smad1/5 and Smad1/5 bands intensity was analyzed and shown.
3. The average relative band intensity of the experiments in (B) were calculated and shown. Vehicle group: n=3, BMP2 group: n=3. The *p value* was based on the student’s t-test. (**P<0.05*)


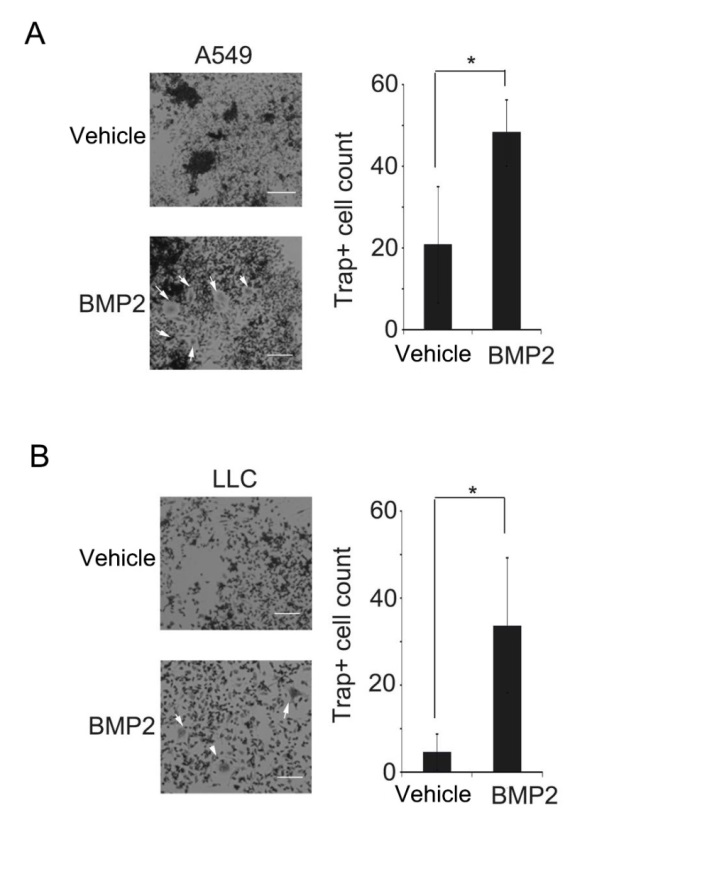


**Fig Sup 3. BMP2 signaling enhanced the differentiation of macrophages into osteoclasts.**

1. 3×10^4^ RAW 264.7 cells were seeded directly into the wells of the 6-well co-culture plates (Corning), and 3×10^4^ MC3T3-E1 cells were seeded into the Corning Cell Culture Inserts with polycarbonate membrane (Transwell^@^, 0.4μm pore size, Corning) of the co-culture 6-well plates. After MC3T3-E1 cells were attached to the membrane of the inserts, 3×10^4^ A549 cells were added on top of MC3T3-E1 cell layer and treated with 20 ng/mL BMP2 or vehicle. TRAP staining was performed for RAW 264.7 cells cultured below on day 6 using a leukocyte acid phosphatase kit. Representative photos were shown. Scale bars, 100μM. Average Trap^+^ cell numbers of at least three fields were shown on the right. The *p value* was based on the student’s t-test. (**P*<0.05, ***P*<0.01)
2. 3×10^4^ RAW 264.7 and 3×10^4^ LLCs were treated as shown in (A). Representative photos were shown. Scale bars, 100μM. Average Trap^+^ cell numbers of at least three fields were shown on the right. The *p value* was based on the student’s t-test. (**P*<0.05, ***P*<0.01)

**
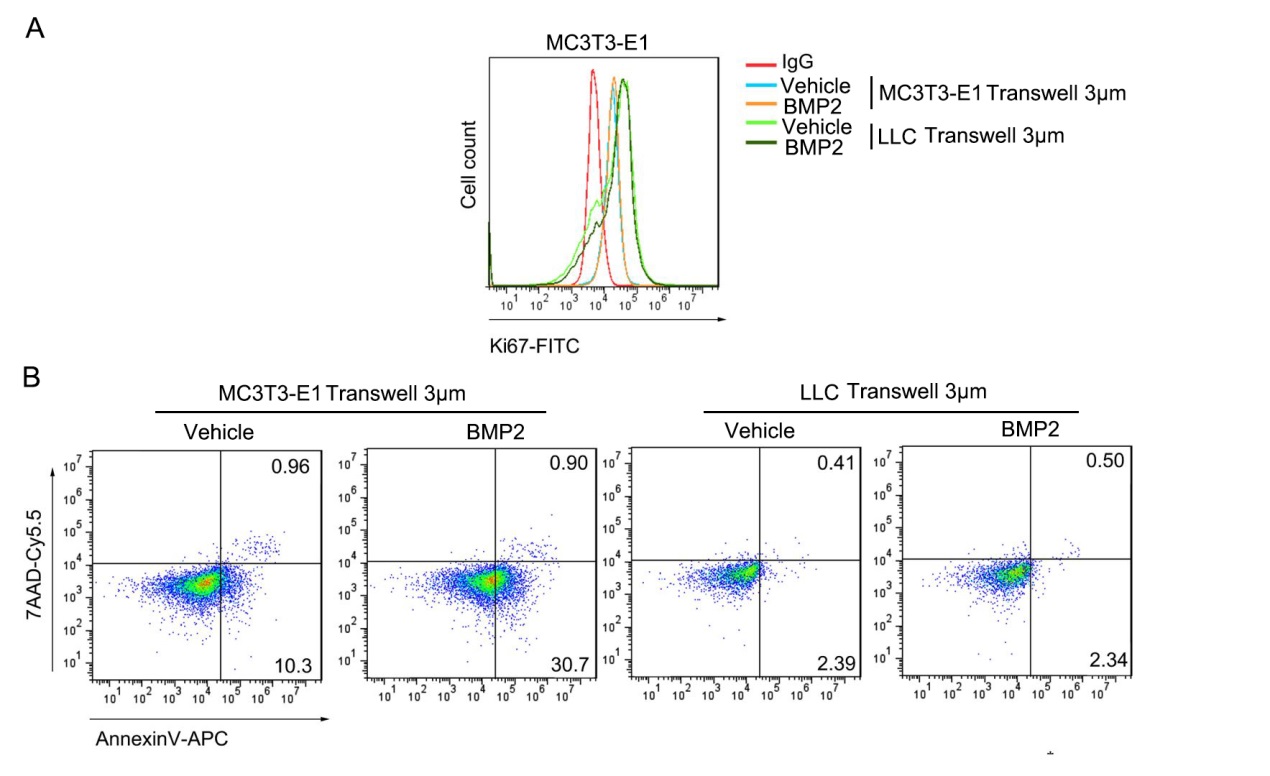
**

**Fig Sup 4. LLCs enhanced the survival of MC3T3-E1 cells independent on BMP2**

1. The expression of Ki67 of MC3T3-E1 cells with different treatment were measured by flow cytometry.
2. Apoptosis of MC3T3-E1 cells with different treatment were measured by flow cytometry via Annexin V and 7AAD double staining.
